# Dosimetric impact of deep learning-based CT auto-segmentation on radiation therapy treatment planning for prostate cancer

**DOI:** 10.1186/s13014-022-01985-9

**Published:** 2022-01-31

**Authors:** Maria Kawula, Dinu Purice, Minglun Li, Gerome Vivar, Seyed-Ahmad Ahmadi, Katia Parodi, Claus Belka, Guillaume Landry, Christopher Kurz

**Affiliations:** 1 Department of Radiation Oncology, University Hospital, LMU Munich, Munich, Germany; 2 Department of Medical Physics, Faculty of Physics, Ludwig-Maximilians-Universität München, Garching, Germany; 3 German Center for Vertigo and Balance Disorders, Ludwig-Maximilians-Universität München, Planegg, Germany; 4 German Cancer Consortium (DKTK), Munich, Germany

**Keywords:** 3D U-Net, Automatic segmentation, Radiation therapy, Prostate cancer, Neural networks, Deep learning

## Abstract

**Background:**

The evaluation of automatic segmentation algorithms is commonly performed using geometric metrics. An analysis based on dosimetric parameters might be more relevant in clinical practice but is often lacking in the literature. The aim of this study was to investigate the impact of state-of-the-art 3D U-Net-generated organ delineations on dose optimization in radiation therapy (RT) for prostate cancer patients.

**Methods:**

A database of 69 computed tomography images with prostate, bladder, and rectum delineations was used for single-label 3D U-Net training with dice similarity coefficient (DSC)-based loss. Volumetric modulated arc therapy (VMAT) plans have been generated for both manual and automatic segmentations with the same optimization settings. These were chosen to give consistent plans when applying perturbations to the manual segmentations. Contours were evaluated in terms of DSC, average and 95% Hausdorff distance (HD). Dose distributions were evaluated with the manual segmentation as reference using dose volume histogram (DVH) parameters and a 3%/3 mm gamma-criterion with 10% dose cut-off. A Pearson correlation coefficient between DSC and dosimetric metrics, i.e. gamma index and DVH parameters, has been calculated.

**Results:**

3D U-Net-based segmentation achieved a DSC of 0.87 (0.03) for prostate, 0.97 (0.01) for bladder and 0.89 (0.04) for rectum. The mean and 95% HD were below 1.6 (0.4) and below 5 (4) mm, respectively. The DVH parameters, V$_{60/65/70\,{\mathrm{Gy}}}$ for the bladder and V$_{50/65/70\,{\mathrm{Gy}}}$ for the rectum, showed agreement between dose distributions within $\pm \, 5\%$ and $\pm \,2\%$, respectively. The D$_{98/2\%}$ and V$_{95\%}$, for prostate and its 3 mm expansion (surrogate clinical target volume) showed agreement with the reference dose distribution within 2% and 3 Gy with the exception of one case. The average gamma pass-rate was 85%. The comparison between geometric and dosimetric metrics showed no strong statistically significant correlation.

**Conclusions:**

The 3D U-Net developed for this work achieved state-of-the-art geometrical performance. Analysis based on clinically relevant DVH parameters of VMAT plans demonstrated neither excessive dose increase to OARs nor substantial under/over-dosage of the target in all but one case. Yet the gamma analysis indicated several cases with low pass rates. The study highlighted the importance of adding dosimetric analysis to the standard geometric evaluation.

## Background

The anatomical structure of the male pelvic region with the prostate surrounded by seminal vesicles, bladder, and rectum, makes modern intensity modulated radiation therapy (RT) a favorable technique for the treatment of localized prostate cancer [1–3]. However, due to variable bladder and rectal filling, random shifts, and deformations of neighboring organs, online adaptation of the treatment plan would be necessary in order to take full advantage of modern radiotherapy techniques [4, 5].

Recontouring of the target volume (TV) and organs at risk (OARs) is an important step in treatment plan adaptation. Previous studies have shown that manual delineation is not only time-consuming (in the order of several minutes) but also prone to inter- and intra-physician variability [6–8].

To address these problems, considerable scientific efforts have been made to develop efficient automatic segmentation tools. Previously, auto-segmentation methods such as (multi)atlas based and hybrid techniques have been considered state-of-the-art [9]. Over time, methods based on convolutional neural networks (CNN) [10] gained more attention [11, 12]. Milletari et al. [13] proposed a 3D fully convolutional neural network architecture trained end-to-end on magnetic resonance (MR) prostate images, referred to as V-Net, and introduced a novel objective function based on the Dice similarity coefficient (DSC). Balagopal et al. [14] presented a hybrid network, having an additional 2D localization network prior to the 3D segmentation network to delineate prostate, bladder, rectum, and femoral heads on pelvic computed tomography (CT) images. In order to overcome the challenges of low soft tissue contrast in CT images as well as blurry boundaries, Wang et al. [15] and Tong et al. [16] focused additionally on edge enhancement techniques. Sultana et al. [17] proposed a two-stage network combining U-Net and generative adversarial network (GAN) architectures [18] for structure localization followed by precise prediction of organ delineation.

Evaluation metrics that are commonly used to measure segmentation performance focus purely on geometric accuracy. The most frequently used are the DSC, the mean, 95%, or maximal Hausdorff distance (HD), the positive prediction value (PPV) or the sensitivity [19]. The two main ideas behind them are: (1) a pixel-wise comparison of ground-truth and predicted segmentation and (2) measuring the distance between the ground-truth and the predicted contours. What carries a higher relevance in clinical practice, however, is the dosimetric accuracy and the quality of the treatment plans that can be achieved on the basis of the predicted segmentations [12, 20]. At the time of writing, no studies exist that have investigated and quantified the dosimetric impact of CT organ delineations for prostate cancer patients obtained from deep CNNs.

In this work a state-of-the-art 3D U-Net architecture for automatic organ segmentation in CT images of low-grade prostate cancer patients was trained. The training was carried out separately for the bladder, prostate, and rectum which are the most important structures for prostate cancer treatment. Since in patients with low-grade prostate cancer, tumorous tissue is located only in the prostate, seminal vesicles were not considered for segmentation. Clinically acceptable VMAT plans were created for all test cases using manual segmentations and the automatic segmentations obtained from the 3D U-Net. This allowed to infer the dosimetric impact of deep learning delineations, which is still rarely present in the literature. The quality of the treatment plans optimized on the automatically generated contours was compared with the reference plans in terms of dose volume-histogram (DVH) parameters, conformity index (CI) and gamma pass rate. In addition, a standard contour-based analysis based on DSC as well as on average and 95th percentile HD calculation was performed. Both, geometric and dosimetric evaluation metrics, were compared in terms of Pearson correlation coefficient to investigate a possible correlation between them.

## Methods

### Database

The dataset used in this study consisted of 69 CT images, along with delineated structures associated with the low-grade prostate cancer treatment performed at the Klinikum Großhadern of the Ludwig Maximilian University (LMU) of Munich. Patients with substantial CT artifacts due to the presence of metal hip implants (1 patient) and fiducial markers (9 patients), causing artifacts throughout the image and especially in the prostate area, were not included in this study. The use of an ultrasound probe for prostate monitoring during irradiation in several cases, did not interfere with CT imaging of the pelvic region, therefore such cases were also included. Similarly, the presence of prostate calcification did not rule out the inclusion of images in the study. CT data have been acquired with a Toshiba Acquilion LB CT scanner (Canon Medical Systems, Japan) using $512\times 512$ pixels in the axial plane and a variable number of slices. Voxel size was $1.074\times 1.074\times 3$ mm$^{3}$. OARs, in particular bladder and rectum, were delineated by a trained radiation oncologist and stored as point clouds (DICOM RT-structs). The prostate contours were redrawn under the supervision of a trained physician according to guidelines for low grade (stage I and II) prostate tumor patients. Using plastimatch [21] images and segmentations were converted from the DICOM RT-struct format, which is required by treatment planning systems and contouring software, into binary masks that are used during the neural network training. Images and binary masks were resampled with the help of nearest neighbor interpolation for masks and linear interpolation for images, to a $1\times 1\times 1$ mm$^{3}$ spaced grid, which was advantageous for the subsequent data augmentation at training stage. While aiming to minimize the influence of contour conversion between the DICOM RT-struct format, defined on a $1.074\times 1.074\times 3$ mm$^{3}$ grid, and binary masks, defined on a $1\times 1\times 1$ mm$^{3}$ grid, we found that employing resampling with nearest neighbor interpolation introduced negligible alterations to the structures. Finally, the dataset has been split into a training, validation, and test sets of 47, 11, and 11 images, respectively. This partitioning was a trade-off between providing enough statistic for testing and validation as well as introducing sufficient variability into the training set.

### 3D U-Net

The 3D U-Net presented here is based on the V-Net architecture [13], developed initially for prostate delineation on MR images. The encoding arm of the network is composed of five levels (including the lowest one) each comprising one (1st level), two (2nd level) or three (3rd–5th levels) convolutional layers and having 16, 32, 64, 128, 256 channels, respectively. The kernel size has been set to $5\times 5\times 5$, stride to $1\times 1\times 1$ and group normalization has been applied after each convolution. The output of a given level is used in the subsequent one as input for the first convolution and is added to the output of the last convolution, thus creating a residual connection. For downsampling between the network levels convolution with a kernel of size $2 \times 2 \times 2$ and stride 2 was used. Throughout the network the PReLU activation was applied. The decoding arm of the 3D U-Net is built in an analogous way, with up-convolution to increase the image size instead. The output of each level of the encoding arm (before the dowsampling) is concatenated with the corresponding input of the decoding arm. The last layer of the network uses the soft-max activation and thresholding of 0.5 to produce two binary masks representing segmentation of the structures and the background. For this project only the segmentation of the structures is relevant.

### Data augmentation

The data augmentation, applied with probability $p_{\mathrm{aug}}$ to each input pair, i.e. image and its segmentation, included 3D rotations around the image center (always aligned with the prostate center of mass), translations, B-Spline-based deformations, and zooming. Translations can be described by three parameters [$x_{\mathrm{trans}}$, $y_{\mathrm{trans}}$, $z_{\mathrm{trans}}$] denoting the maximal translation distances along each axis. Similarly, Euler rotations can be denoted by the maximal rotation angles [$\alpha$, $\beta$, $\gamma$] around the superior-inferior, anterior-posterior and medial-lateral axis, respectively. Zooming re-sizes each axis by a factor randomly drawn from [$l_{{\min }}$, $l_{{\max }}$]. The pixel intensities have been truncated to fit the soft tissue window [$I_{{\min }}$, $I_{{\max }}$] and subsequently rescaled to [− 1, 1]. The deformation field is defined on a grid of $n \times n \times n$ control points with random shifts drawn from a Gaussian distribution [$\mu$, $\sigma$]. In the last step of the augmentation pipeline, a central part of each image has been cropped to 128$\times$128$\times$128 due to memory limitations on the GPU. Nevertheless, the clinically relevant high dose regions close to the prostate were not affected by the cropping. While setting the initial values for the data augmentation parameters, special care was taken not to introduce strong artifacts or create unrealistic deformations.

### Training

Training on single-label data has been performed separately for three regions of interest: prostate, rectum, and bladder. Each model has been trained on an NVIDIA Quadro P6000 GPU with the Keras implementation of the Adam optimizer ($\beta _{1}=0.9,\ \beta _{2}=0.999,\ \epsilon =1e-07$) and the Dice loss function applied to both, segmentations and the background. The set of hyper-parameters to be optimized can be divided into two sub-groups: data augmentation related parameters such as maximal translation shifts, rotation angles, zooming and soft-tissue window limits, B-Spline deformation parameters, augmentation probability and training related parameters such as the learning rate and number of epochs. The optimization of the hyper-parameters was performed via a random search. Training with a certain set of hyperparameters was performed until the loss function evaluated on the validation data did not decrease further for several dozen epochs.

### Treatment planning

For all test cases, single arc photon VMAT treatment plans were generated using a research version of the commercial treatment planning system (TPS) RayStation (version 8.99, RaySearch, Sweden). All plans aimed at a total dose of 74 Gy in 37 fractions. The generic beam model of an Elekta Synergy Linac (Elekta, Sweden) with Agility multi-leaf-collimator was used. For each test case, two treatment plans were optimized on the same planning CT image, one based on the expert segmentation and one based on the 3D U-Net segmentation of rectum, bladder, and prostate. In both scenarios, in accordance with our facility’s clinical guidelines, a PTV margin of 6 mm (posterior 5 mm) was applied around the prostate. The same optimization settings, i.e., the same objectives and weights for planning target volume (PTV), bladder, and rectum, for both manual and automatic segmentation were used. Settings were chosen using the expert segmentation such that a PTV coverage of at least V$_{95\%}=100\%$ was achieved (no normalization was applied after optimization), while dose to OARs was below the recommendations of the QUANTEC report [22]. Since the dose optimization problem does not have a unique solution, calculation outcomes might be different, despite using highly similar sets of contours. In order to perform a dosimetric evaluation that captures differences in dose distributions caused primarily by variations in the delineated structures and not by the solution ambiguity of the optimization problem, care was additionally taken to choose optimization settings that produce consistent planning results by applying small perturbations to the manual segmentation. For this, the original RT-structs were converted to binary masks and back to DICOM RT-structs. Then a new plan was generated with the same optimization settings and dosimetrically compared to the initial plan using the original RT-structs. With the final parameters (see weights in Table 1) dose distributions for all test cases were achieved that deviated less than $\pm \, 2\%$ in the considered OAR and target DVH parameters (see following section) but were not statistically significant. For all test patients and all calculated dose distributions, the ICRU Report 83 guidelines concerning the PTV [23], i.e. D$_{98\%} \ge 95\% \mathrm{\ of\ the\ prescribed\ dose}$ and D$_{2\%} \le 107\% \mathrm{\ of\ the\ prescribed\ dose}$, were met as well. These settings were then used to optimize treatment plans using the 3D U-Net segmentations without further user interaction. Table 1 summarizes the goals of the treatment planning along with the importance of each factor.

**Table 1:** Clinical goals used in the TPS RayStation for VMAT plan generation

Function	ROI	Description	Weight
Max dose	Rectum	74 Gy	0.03
Max EUD, A = 12	Rectum	64 Gy	0.11
Max EUD, A = 8	Bladder	63 Gy	0.03
Min dose	PTV	74 Gy	0.42
Uniform dose	PTV	74 Gy	0.07
Max dose	PTV	77.7 Gy	0.21
Dose fall-off	External	[H]74 Gy, [L] 10 Gy,	0.13
		Low dose distance 1 cm	

For each region of interest (ROI) a given objective function was assigned. Weights were normalized to 1 and indicate the importance of each parameter during plan optimization

### Data evaluation

In order to evaluate the network-generated contours, DSC, average HD and 95% HD (defined as 95th percentile of the distances between boundary points), have been calculated for all test cases with expert delineations as the reference ground truth. Since there is no clear boundary between the rectum and colon, evaluation of the network predictions was limited to the slices containing the ground truth segmentation, i.e. no additional penalty was applied for colon misclassification. Apart from that, geometric data evaluation (DSC, HD$_{\mathrm{avg}}$, and HD$_{\mathrm{95\%}}$) has been restricted to the $128\times 128\times 128$ volume.

The dose distributions for predicted and ground truth contours were analyzed using a 3D global gamma-criterion with a pass-rate of (3%, 3 mm), where only voxels with at least 10% of the prescribed dose were considered. Additionally, CI defined by Paddick [24] was calculated. This index has an ideal value of one and plan quality decreases with decreasing index value. Both dose distributions were also compared in terms of clinically relevant target and OAR DVH parameters. For prostate and its 3 mm expansion (surrogate CTV), values of D$_{98\%}$ , D$_{2\%}$ and V$_{95\%}$ were determined. Similarly, for the rectum V$_{50/65/70\,{\mathrm{Gy}}}$ and for the bladder V$_{60/65/70\,{\mathrm{Gy}}}$ were calculated. All DVH parameters were determined using the ground truth segmentations and the dose distributions optimized either on the predicted or on the ground truth contours. To assess the statistical differences between DVH parameters for plans optimized on the manually and the U-Net generated contours, a Wilcoxon signed-rank test with a statistical significance threshold of $p=0.05$ was used.

To investigate the correlation between the dosimetric and geometric metrics, the Pearson correlation coefficient [25] between (1) DSC of prostate and gamma index, (2) average DSC and gamma index, and (3) DSC and DVH parameters were calculated.

## Results

### Hyperparameter optimization

The following values of hyperparameters have lead to satisfactory results: $p_{\mathrm{aug}}=0.93$, rotation angles $\alpha =20^{\circ }$, $\beta =\gamma =10^{\circ }$, translation shifts $x_{\mathrm{trans}}=y_{\mathrm{trans}}=z_{\mathrm{trans}}=10\,{\mathrm{mm}}$, $l_{{\min }}=0.9$, $l_{{\max }}=1.1$, $I_{{\min }}=-150 \,{\mathrm{HU}}$, $I_{{\max }}=150 \,{\mathrm{HU}}$, grid control points $n \times n \times n = 15\times 15\times 15$, $\mu =0$, $\sigma = 30$. After 20k epochs with a batch size of two, we found all the loss functions to converge with no signs of overfitting. The learning rate of $10^{-3}$ has been shown to perform best.

### Contour-based analysis

**Fig. 1:**
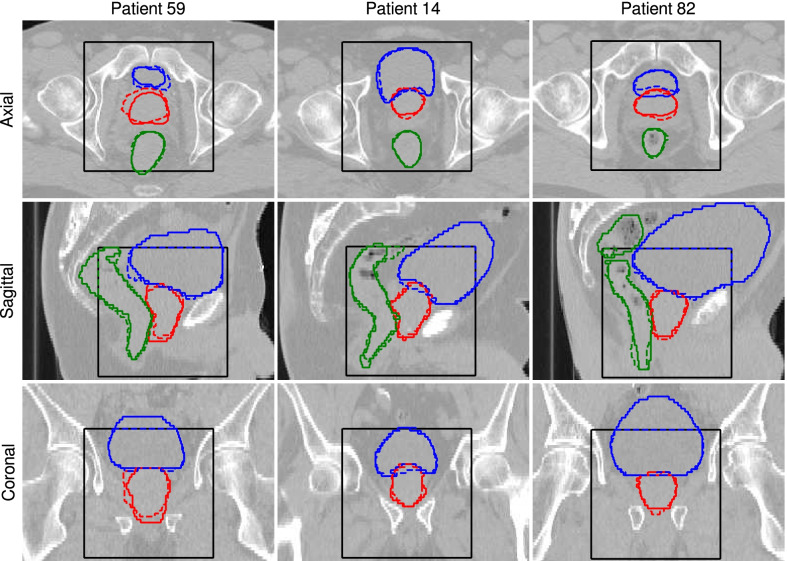
Axial, sagittal, and coronal slices showing (solid lines) the ground truth contours and (dashed lines) predictions generated by the 3D U-Net. (Red) prostate, (green) rectum, and (blue) bladder delineations are presented for three test patients showing (left) the worst, (middle) closest to the average, and (right) the best agreement with the ground truth by means of DSC for prostate. The black box indicates the region where the contours were predicted by the U-net

Figure 1 illustrates ground truth and automatically-generated delineations of prostate, rectum, and bladder for three test patients. Images with the best, closest to the average, and the worst values of DSC for prostate are displayed.

Table 2 collects the results of the geometric analysis for all test patients. Mean DSCs (standard deviation) of 0.87 (0.03), 0.97 (0.01), 0.89 (0.04) were achieved for the prostate, bladder, and rectum, respectively. The highest average DSC value was observed for the bladder, which can be attributed to its relatively large size. A slightly worse performance has been observed for rectum and subsequently prostate. The values of the average HD were 1.6 (0.4) mm, 0.95 (0.2) mm, 1.4 (0.7) mm for prostate, bladder, and rectum, respectively. The values of the 95% HD show the same trend 4 (1) mm, 2.5 (0.5) mm, 5 (4) mm for prostate, bladder, and rectum, respectively.

**Table 2:** Contour based metrics: DSC, average Hausdorff distance (HD$_{\mathrm{avg}}$) and 95% Hausdorff distance (HD$_{\mathrm{95\%}}$) of all test patients

	DSC			(HD$_{\mathrm{avg}}/_{95\%}$) (mm)		
	Prostate	Bladder	Rectum	Prostate	Bladder	Rectum
Pat. 11	0.90	0.96	0.90	1.4/4.5	1.0/2.3	1.2/4.0
Pat. 14	0.88	0.96	0.88	1.5/3.6	1.0/3.6	1.2/3.5
Pat. 27	0.86	0.97	0.91	1.5/3.7	0.9/2.2	1.1/3.0
Pat. 32	0.87	0.96	0.78	2.2/5.1	1.1/3.2	3.4/14.9
Pat. 43	0.85	0.94	0.90	1.7/4.2	1.5/3.2	1.2/3.3
Pat. 44	0.88	0.96	0.92	1.3/3.6	0.8/2.1	0.8/2.2
Pat. 52	0.83	0.97	0.92	2.0/5.5	0.9/2.3	1.4/3.5
Pat. 59	0.82	0.97	0.90	2.3/6.2	0.9/2.6	1.3/4.9
Pat. 81	0.91	0.97	0.87	1.2/3.4	0.9/2.1	1.8/8.3
Pat. 82	0.92	0.97	0.88	1.0/2.3	0.8/2.1	1.2/3.5
Pat. 90	0.85	0.97	0.91	1.6/4.3	0.8/2.1	1.0/2.9
Mean (STD)	0.87 (0.03)	0.97 (0.01)	0.89 (0.04)	1.6 (0.4)/4 (1)	0.95 (0.2)/2.5 (0.5)	1.4 (0.7)/5 (4)

The last row presents the mean and standard deviation (STD) over all test cases

### Dosimetric analysis

**Fig. 2:**
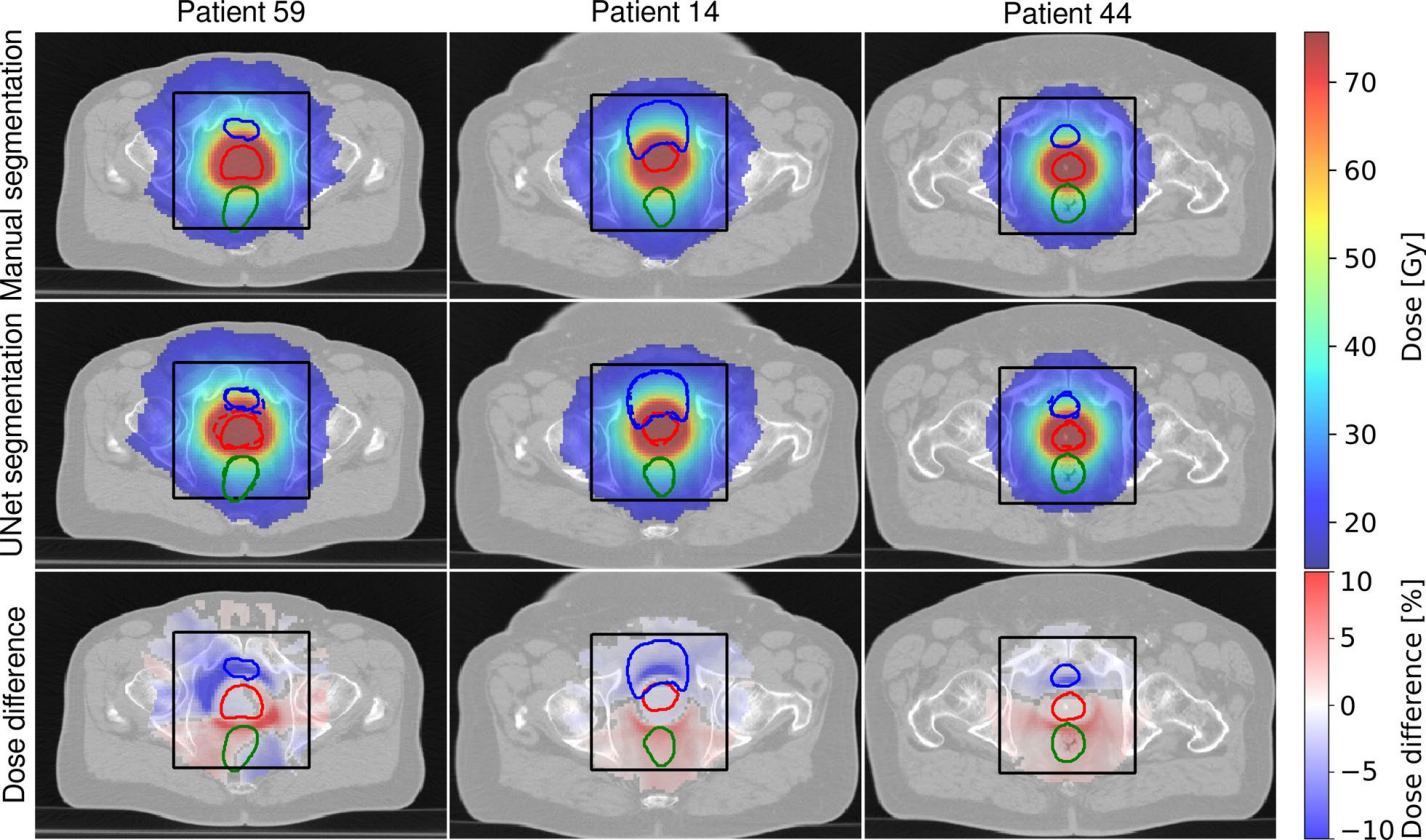
Dose distributions on axial slices of three test patients showing (left) the worst, (middle) the average and (right) the best agreement quantified by the gamma-index of the treatment plan optimized on (top) the manual segmentation and (middle) the 3D U-Net segmented images. Additionally, relative dose differences are presented. For improved visibility, dose below 25% of the dose prescribed to PTV and deviations below 0.4% on the difference plot are not displayed. Ground truth contours of (green) rectum, (blue) bladder, and (red) prostate, are also shown. The black box indicates the region where the contours were predicted by the U-Net

**Fig. 3:**
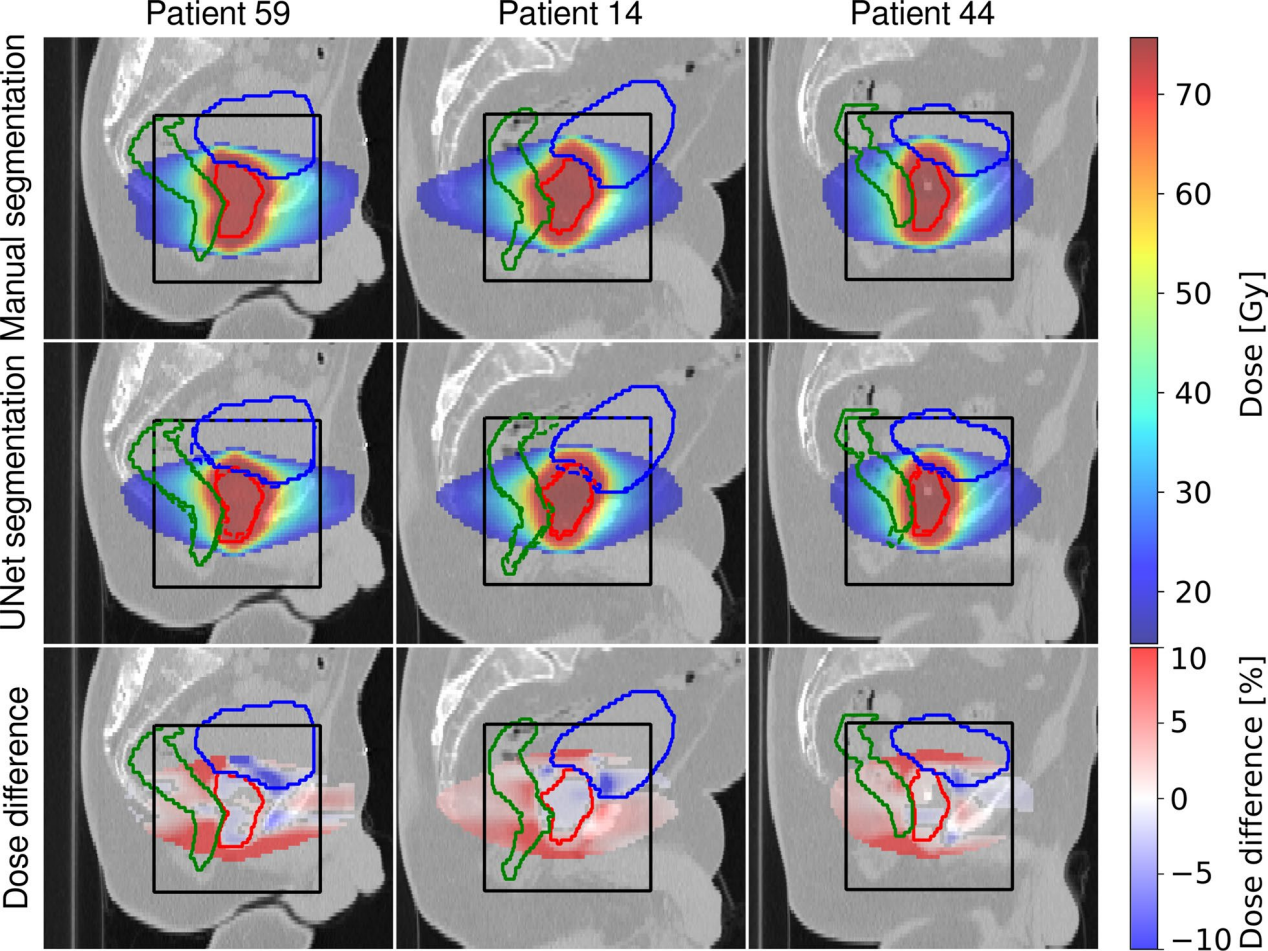
Dose distributions on sagittal slices of three test patients showing (left) the worst, (middle) the average and (right) the best agreement quantified by the gamma-index of the treatment plan optimized on (top) the manual segmentation and (middle) the 3D U-Net segmented images. Additionally, relative dose differences are presented. For improved visibility, dose below 25% of the dose prescribed to PTV and deviations below 0.4% on the difference plot are not displayed. Ground truth contours of (green) rectum, (blue) bladder and (red) prostate are also shown. The black box indicates the region where the contours were predicted by the U-Net

**Fig. 4:**
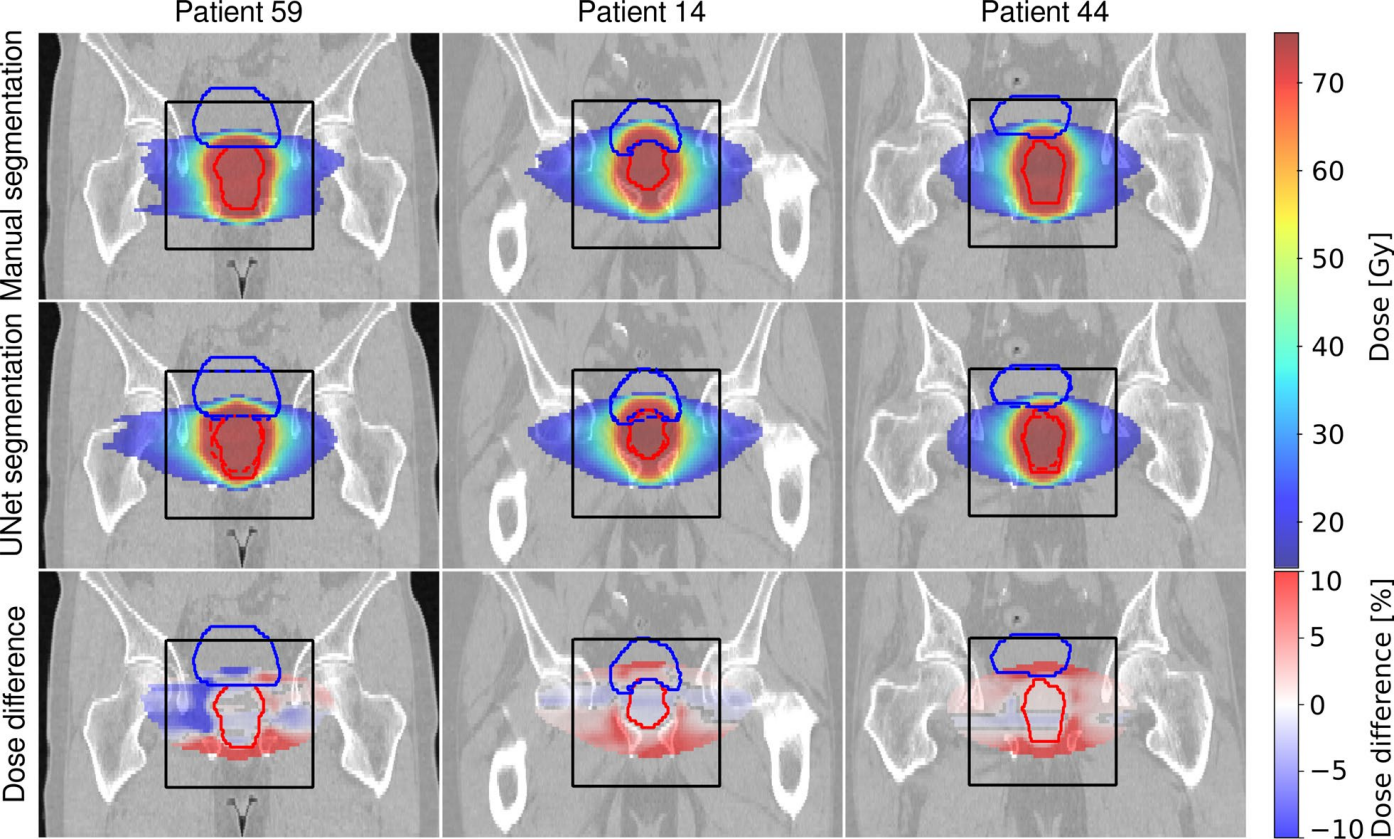
Dose distributions on coronal slices of three test patients showing (left) the worst, (middle) the average and (right) the best agreement quantified by the gamma-index of the treatment plan optimized on (top) the manual segmentation and (middle) the 3D U-Net segmented images. Additionally, relative dose differences are presented. For improved visibility, dose below 25% of the dose prescribed to PTV and deviations below 0.4% on the difference plot are not displayed. Ground truth contours of (green) rectum, (blue) bladder and (red) prostate are also shown. The black box indicates the region where the contours were predicted by the U-Net

Figures 2, 3 and 4 illustrate dose distributions of three exemplary patients with the highest, the average, and the lowest gamma pass-rate in axial, sagittal and coronal views. The reference dose distribution optimized using the ground truth contours, the 3D U-Net dose distribution optimized using the predicted delineations, and their difference are shown. Deviations from the reference plan were found to be in the range of $\pm \,10\%$ and were located primarily outside of the prostate. The largest differences were found close to the borders of the PTV region, where dose gradients are steep (6 mm away from the prostate boundary).

The quantitative results of the dosimetric comparison are summarized in Table 3. The value of the CI for the reference plans is in the range of 0.81 and 0.89 with an average (standard deviation) of 0.85 (0.03). For the plans calculated on 3D U-Net generated contours the CI is in the range of 0.69 and 0.88 with an average of 0.78 (0.06). The gamma-pass rates (3 mm, 3%) were between 71 and 94%, with an average value of 85%.

**Table 3:** Gamma pass rate (3 mm, 3%) and conformity index calculated for plans optimized on manual (CI$_{\mathrm{man}}$) and 3D U-Net (CI$_{\mathrm{3D U-Net}}$) generated segmentations of all test patients

	CI$_{\mathrm{man}}$	CI$_{\mathrm{3D U-Net}}$	(3 mm, 3%) (%)
Pat. 11	0.87	0.78	91
Pat. 14	0.83	0.83	89
Pat. 27	0.83	0.75	88
Pat. 32	0.89	0.77	79
Pat. 43	0.83	0.78	93
Pat. 44	0.82	0.88	94
Pat. 52	0.84	0.69	77
Pat. 59	0.87	0.70	71
Pat. 81	0.88	0.82	87
Pat. 82	0.85	0.85	92
Pat. 90	0.81	0.72	74
Mean	0.85 (0.03)	0.78 (0.06)	85 (8)

**Fig. 5:**
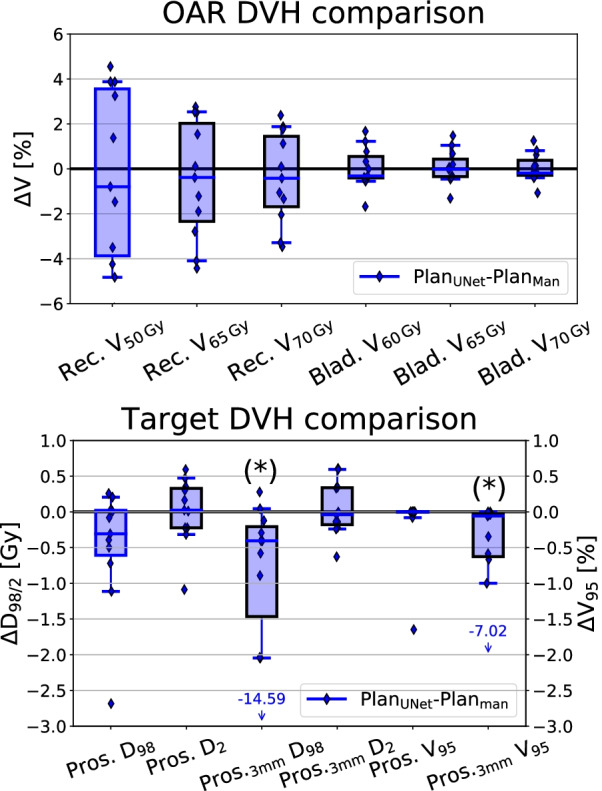
Differences between the two treatment plans optimized using U-Net- and manually-generated contours. DVH parameters for (top) OARs (bladder and rectum) and (bottom) TV (prostate, prostate + 3 mm margin). Each patient is represented by a single data point, while whiskers indicate the 5th–95th percentile. Dose values correspond to the total dose of 74 Gy delivered during fractionated treatment. Asterisks indicate the statistically significant differences

Figure 5 illustrates differences between clinically relevant DVH parameters of the two optimized dose distributions, evaluated on the reference, i.e. manually delineated, contour set. Again, the reference dose distribution was optimized using the ground truth delineations and compared the the dose distribution optimized on the 3D U-Net predicted contours. For rectum and bladder, all the differences are below 5% and 2%, respectively. None of them has been found to be statistically significant ($p\ge 0.05$). No clear trend of increased or decreased bladder and rectum dose for the 3D U-Net segmentation-based plans was found. Similarly, differences for the target volume are mostly below 3 Gy/2% for D$_{98}$, D$_{2}$ and V$_{95}$, apart for one outlier (patient 59, 10% of the test set) where the network struggled to delineate the prostate, which is also reflected in the relatively low DSC of 0.82 and gamma index of 71%. The only statistically significant differences have been found for the surrogate CTV for D$_{98}$ and V$_{95}$. No tendencies for the D$_{2}$ parameter have been observed, but the 3D U-Net based plans tend to have reduced values of D$_{98}$ and V$_{95}$ for both, prostate and its 3 mm expansion, indicating a slight reduction of target coverage which is in line with the reduced CI values.

### Pearson correlation coefficient

The Pearson correlation coefficient with the *p* value for the DSC of prostate and gamma index was 0.67 ($p=0.023$), which shows a moderate positive correlation. No statistically significant results were obtained for the other parameters.

## Discussion

In this work a 3D U-Net has been successfully trained and applied for CT-based organ segmentation in the male pelvic area. The evaluation of the network’s performance was based not only on the commonly used geometric metrics, but also on clinically relevant dosimetric parameters.

Satisfactory performance was observed with regard to the geometric accuracy of the contour delineation, indicating a high degree of similarity between automated and manual segmentations. The best results were observed for bladder segmentation, followed by the rectum, and prostate. The best values of DSC and HD for the bladder can be explained firstly, by its simple geometry and secondly, by its relatively large size, which makes an incorrect prediction of a group of edge pixels less relevant with regard to the correctly classified central part of this organ. The low contrast of the prostate on the CT images makes its segmentation most challenging, which was reflected in a DSC of 0.87. With the exception of one case (Pat. 32) in which a substantial portion of the colon was misclassified as part of the rectal contour, the rectum segmentation showed a relatively high dice equal to 0.87. Since the rectum-colon boundary is visually difficult to identify and is not located in the high dose region, we decided to reduce the penalty for this type of misclassification during the final evaluation (testing) by truncating the volume of interest to the axial slices that contained the ground truth segmentation.

Quantitative test outcomes showed state-of-the-art network performance in terms of DSC, mean and 95% HD. The 2D–3D hybrid network for localization and subsequent organ segmentation proposed by Balagopal et al. [14] achieved a DSC of 0.9 for prostate, 0.95 for bladder and 0.84 for rectum. The edge-calibrated multitask network by Tong et al. [16] showed an overall bladder, rectum, and prostate segmentation performance of DSC = 0.89. The UNet-GAN hybrid architecture by Sultana et al. [17] achieved DSC = 0.90 for prostate. A more detailed comparison is shown in Table 4. In all studies, bladder achieved the highest segmentation accuracy, followed by prostate and rectum.

**Table 4:** Quantitative comparison of geometric metrics with state-of-the-art segmentation algorithms

	Present work	Balagopal et al. [14]	Sultana et al. [17]	Tong et al. [16]
*Prostate*				
DSC	0.87 ± 0.03	0.90 ± 0.02	0.90 ± 0.05	0.86 ± 0.06
HD$_{\mathrm{avg}}$	1.6 ± 0.4	–	1.56 ± 0.37	1.01 ± 0.65
HD$_{\mathrm{95\%}}$	4 ± 1	–	5.21 ± 1.2	3.51 ± 1.66
*Bladder*				
DSC	0.96 ± 0.01	0.95 ± 0.02	0.95 ± 0.02	0.96 ± 0.02
HD$_{\mathrm{avg}}$	0.95 ± 0.2	–	0.95 ± 0.15	0.97 ± 0.53
HD$_{\mathrm{95\%}}$	2.5 ± 0.5	–	4.37 ± 0.56	3.17 ± 3.61
*Rectum*				
DSC	0.89 ± 0.04	0.84 ± 0.04	0.84 ± 0.04	0.86 ± 0.07
HD$_{\mathrm{avg}}$	1.4 ± 0.7	–	1.78 ± 1.3	1.22 ± 1.05
HD$_{\mathrm{95\%}}$	5 ± 4	–	6.11 ± 1.5	4.34 ± 5.30

In the current work, 1 patient with a metal hip implant and 9 patients with fiducial markers were excluded from the study due to artifacts. Applying the trained network to these cases resulted in a DSC of 0.60 (7) for prostate and average Hausdorff distance of 32.5 (8) mm, demonstrating that the trained network cannot be used for images with such artifacts. The available 10 cases are neither sufficient to train a separate model nor to expect a visible effect on the training in combination with the other training data-sets (several images would also have to be set aside for validation and testing, further reducing the training dataset). A potential solution to this issue could be collecting a larger database of images with artifacts and carrying out an independent training.

The ground truth bladder and rectum segmentations were assembled over a course of 2.5 years at the LMU Klinikum and originated from several physicians. In contrary, prostate segmentation has been re-drawn for the purpose of this study. Multi-observer contours in the training set might be seen as an advantage, as the network learns how to generalize and does not adjust to the contouring style of one physician only. On the other hand this might lead to lower testing outcomes, since the network predictions compared against contours drawn by different physicians will be ranked differently. This also sets an upper limit on the network performance measured by means of geometric metrics which is in the order of the expectable inter-observer differences [26].

Due to GPU memory limitations, images were cropped around the prostate center of mass, causing truncation of bladder and rectum parts in some cases. On the one hand, this could have made it easier to predict the outer walls, on the other hand, this reduced the organ volume. Since these factors have the opposite effect on DSC and are small in themselves, the effect on DSC is deemed negligible, while the value of HD might have been slightly underestimated. The truncated sections were always located in the low dose region and therefore dosimetric analysis and plan optimization were not affected.

In the scope of the additional dosimetric analysis, target volume D$_{98}$, D$_{2}$ and V$_{95}$ of the plans optimized using 3D U-Net contours were found to differ only slightly from the reference plans based on expert delineations, however a trend of lower D$_{98}$ and V$_{95}$ was observed as shown in Fig. 5. In only one case (patient 59), major deviations, i.e. D$_{98}=-\,14.59$ Gy and V$_{95}=-\,7.02\%$ for surrogate CTV, were observed. This can be attributed to an incorrect prostate contouring that is shifted towards the bladder, as can be seen in Fig. 1.

The average value of the CI was 0.78 (0.06) for the plans optimized on 3D U-Net generated contours and 0.85 (0.03) for reference plans. The lower value of the average CI confirms slightly worse target coverage. The treatment plans derived from automatic contours yielded lower CI since the evaluation was performed using the ground truth contours. In contrary, the reference plans have been optimized and evaluated on the same set of contours, and are thus biased towards higher values by design.

Due to the lack of an absolute reliability of the automatic segmentation, human review is still unavoidable. Nonetheless, introducing a method that has a potential to accelerate the contouring process in the majority of cases, as it was show in [27] or in a similar study considering lung cancer patients [28], would be an improvement with respect to current clinical practice.

Analysis of DVH parameters for rectum showed that treatment plans optimized on 3D U-Net-generated contours did not result in statistically significant differences measured by V$_{50/65/70\ {\mathrm{Gy}}}$. No statistically significant differences were found for the bladder as well. Results indicate that plans optimized on automatically generated contours do not overdose the neighboring OARs, i.e. bladder and rectum.

The gamma index analysis resulted in pass rates of 71–94% with a mean value of 85%. The most prominent differences between dose distributions have been detected close to the PTV border. The degree of the discrepancies correlates closely with the discrepancies between PTV borders (ground truth and predicted) as steep dose gradients are desirable during dose optimization. Thus, the main organs affected by these differences were the bladder and the rectum, for which the most relevant DVH indices have been carefully analyzed in this study. Inside the PTV we did not observe any ‘hot-spots’ exceeding 107% of the prescribed dose. We also did not notice any consistent dose clustering outside of the PTV. The maximum dose delivered to femoral heads was always below 35 Gy, which is significantly lower than the recommended threshold of 50 Gy.

The only statistically significant correlation was found between the DSC of the prostate and the gamma index. The Pearson coefficient showed a moderately positive correlation only. No statistically significant correlation was found between the gamma pass-rate and the DSC values of OARs and between the DVH parameters and the DSC. On the contrary, we have observed that it is not uncommon for patients to show a very similar DSC for the prostate, which is the most important segmentation in relation to the treatment planning of prostate cancer, while showing a very different gamma pass-rate e.g. DSC$_{\mathrm{Pat.43}}$ = DSC$_{\mathrm{Pat.90}}$ = 0.85 while $\gamma _{\mathrm{Pat.43}}=93$ and $\gamma _{\mathrm{Pat.90}}=74$ or DSC$_{\mathrm{Pat.44}}$ = 0.88, DSC$_{\mathrm{Pat.81}}$ = 0.91 while $\gamma _{\mathrm{Pat.44}}=94$ and $\gamma _{\mathrm{Pat.81}}=87$. This leads to the conclusion, that a high geometric similarity between contours, commonly evaluated by the means of DSC, does not necessarily result in a high fidelity dose distribution optimized using these contours. Since eventually, the dosimetric analysis is clinically more relevant the results of this study highlight that the latter should always be carried out in addition to the geometric analysis.

Another important factor to consider is the contour conversion between two formats: the point cloud format (DICOM RT-Struct) required by the contouring software as well as the TPS, and the binary masks required for CNN training. The use of nearest neighbors interpolation in the conversion pipeline did not introduce any noticeable differences during structure conversion.

One possible improvement to this study could be to prepare separate training images for the bladder and rectum by cropping images around their mass centers and adjusting the soft tissue window to match closer their HU range. This could help create more precise contours, but should not significantly affect the dosimetric analysis as the parts of the OAR structures relevant for treatment planning are located in close vicinity of the prostate, which was used as center for cropping in this study. Furthermore, prostate patients with tumor stages III and IV could be included in future studies by including seminal vesicles in the prostate contour or training a separate network. However, this is a challenging task since in clinical practice the CTV/PTV might contain different proportions of seminal vesicles depending on the exact tumor stage. Therefore, the CTV/PTVs including the seminal vesicles might have more pronounced variations between patients and thus more training data would be required.

## Conclusions

A 3D U-Net was successfully trained for organ segmentation on CT images of the male pelvic region. The geometric accuracy measured with DSC, mean and 95% HD showed state-of-the-art performance of our algorithm. Analysis based on clinically relevant DVH parameters of VMAT plans did not show excessive dose enhancement to OARs and proved sufficient for treatment target volume coverage in nine out of ten cases. Nevertheless, the gamma pass rate was not always acceptable, indicating that human review is crucial. No strong statistically relevant correlation between geometric and dosimetric metrics was observed, suggesting that both types of analysis should be included in the evaluation of automatic organ segmentation in the scope of radiotherapy.

## Data Availability

The patient data will not be available due to missing ethics approval for public sharing. V-Net code: https://github.com/faustomilletari/VNet.

## Abbreviations

3D U-Net: 3 dimensional U-Net architecture
CI: Conformity index
CNN: Convolutional neural network
CT: Computed tomography
CTV: Clinical target volume
DICOM: Digital imaging and communications in medicine
DSC: Dice similarity coefficient
DVH: Dose-volume histogram
GAN: Generative adversarial network
GPU: Graphics processing unit
HD: Hausdorff distance
HD_avg_: average Hausdorff distance
95% HD or HD_95%_: 95th percentile Hausdorff distance
HU: Hounsfield unit
RT: Radiation therapy
MR: Magnetic resonance
OAR(s): Organ(s) at risk
PPV: Positive prediction value
PReLU: Parametric rectified linear unit
PTV: Planning target volume
TPS: Treatment planning system
TV: Target volume
VMAT: Volumetric Modulated Arc Therapy
